# Peak Oxygen Uptake and Exercise Capacity of Children Undergoing Leukemia Treatment

**DOI:** 10.3390/ijerph17238732

**Published:** 2020-11-24

**Authors:** Aleksandra Kowaluk, Marek Woźniewski

**Affiliations:** Department of Physiotherapy in Surgical Medicine and Oncology, Faculty of Physiotherapy, University School of Physical Education in Wroclaw, 51-612 Wroclaw, Poland; marek.wozniewski@awf.wroc.pl

**Keywords:** cardiorespiratory fitness, children, leukemia, physical activity, morphotic parameters, blood

## Abstract

The aim of the study was to assess the exercise capacity (VO_2peak_) of children undergoing leukemia treatment and to compare the results with healthy children. Furthermore, we assessed the influence of treatment methods on the level of exercise capacity and the increase in sedentary behaviors. The study comprised 21 children (12 boys and 9 girls) undergoing treatment for acute lymphoblastic leukemia (ALL) (*n* = 13) and acute myeloid leukemia (AML) (*n* = 8). The subjects were aged 7–13 years (mean age 10.7, SD 2.0 years). Cardiorespiratory fitness was assessed by using the ergospirometry test. Progressive Godfrey protocol was performed. The level of physical activity was assessed by using the questions from the Health Behavior in School-Aged Children (HBSC 2018) questionnaire. The study results showed that children undergoing leukemia treatment were characterized by a reduced level of exercise capacity. The measured value of VO_2peak_ in the group of treated children was, on average, 22.16 mL·kg^−1^·min^−1^. The mean values of VO_2peak_ predicted for this age group were 45.48 mL·kg^−1^·min^−1^ (SD, 3.8). The measured value of VO_2peak_ in the study group with the division into age groups was, on average, 21.21 mL·kg^−1^·min^−1^ in the group of children aged 7–10 years. In the group of children aged 11–13 years, this parameter was 22.64 mL·kg^−1^·min^−1^. Lack of physical activity and failure to meet the standards for the minimum level of weekly physical activity (MVPA index—moderate-to-vigorous physical activity) probably contribute to the deterioration in exercise capacity level of cancer-treated children.

## 1. Introduction

Leukemia is one of the most common childhood cancers, and the effectiveness of its treatment is steadily increasing. However, cancer therapy is aggressive, and it leads to many side effects that occur during treatment [1,2] and many years after its completion [3]. One of the most common complications of cancer treatment is a large decrease in morphotic parameters of blood. Low blood counts, especially the decrease in hemoglobin concentration, are the reason of insufficient oxygen transport to the working muscles and other tissues [4,5]. Anemia and reduced blood perfusion in skeletal muscles are the causes of insufficient oxygen supply during physical effort, which leads to a reduction in cardiorespiratory fitness that manifests by decreased exercise tolerance [6]. As a result, children who undergo cancer treatment reduce their daily physical activity and prefer a sedentary lifestyle [7]. Such habits and a reduced level of exercise capacity can persist in adult childhood-cancer survivors [8].

Intensive anticancer therapy leads to a significant decrease in exercise capacity parameters in children, which was confirmed by San Juan et al. Peak oxygen uptake values (VO_2peak_) in the group of children with acute lymphoblastic leukemia (ALL) undergoing the maintenance phase of the treatment were significantly lower when compared to the group of healthy peers (25.3 ± 6.5 mL·kg^−1^·min^−1^ vs. 31.9 ± 6.8 mL·kg^−1^·min^−1^) [9]. These results confirm the essence of the treatment-induced problem with cardiorespiratory fitness impairment and the insufficient implementation of rehabilitation methods to combat the problem of inactivity. It has also been shown that the improvement in exercise capacity parameters increases the survival rate, while reduced exercise capacity already at the level of 3.5 (mL·kg^−1^·min^−1^) contributes to the decrease in survival rate by 12% [10].

During anticancer therapy, children often participate in rehabilitation activities. However, it is primarily aimed at relieving the symptoms of treatment, such as reducing contractures and muscle atrophy. Children who undergo treatment are not included in a comprehensive program. The rehabilitation is often aimed only at reducing patients’ temporary problems. Exercise is rarely used to keep children physically active and ensure at least a moderate level of exercise capacity. It is crucial to implement aerobic exercise programs at the very beginning of treatment, to prevent an excessive decrease in VO_2peak_ [11].

There are many studies that confirm the beneficial effects of aerobic exercise programs in the group of children undergoing cancer treatment. They can also prevent the development of long-term deficits in cardiorespiratory fitness and peak oxygen values if they are implemented early [12]. The measurement of the baseline peak oxygen uptake at the beginning of the treatment allows for the assessment of the cardiorespiratory fitness and the choice of an individual aerobic exercise program. Periodic follow-up and monitoring of the cardiorespiratory parameters allow the modification of the individual rehabilitation program to the current condition of children [13]. It is important to compare the level of exercise capacity of treated children with the results of healthy children. This will show how the extent of cancer disease has reduced the capacity level of children undergoing leukemia treatment.

The Cardio-Pulmonary Exercise Test (CPET) is a valuable tool. However, it is relatively rarely used in oncology clinics due to costs, the need for special equipment for respiratory gas analysis, and trained personnel [14]. CPET is one of the methods used to assess exercise tolerance, which reflects the level of cardiorespiratory fitness. This test is the most reliable and commonly used tool to measure the peak oxygen uptake value using respiratory gas analysis [15]. This measurement is considered by the World Health Organization to be the gold standard for assessing aerobic fitness [16]. Peak oxygen uptake is regarded as the most objective and reliable parameter for assessing exercise capacity. CPET results provide clinically useful information and are helpful in visualizing the body’s physiological responses to a given physical effort [15,17]. This test performed in children during leukemia treatment is also a useful additional measurement for assessing the severity of cardiopulmonary disorders [18]. The results of these clinical trials may be useful in creating new, or improving the already existing, models of rehabilitation in children during treatment. Such models should prevent the excessive decline in exercise capacity among children both during and after treatment.

The aim of the study was to assess the peak oxygen uptake in children undergoing leukemia treatment and to compare the results with the group of healthy children. Furthermore, we assessed the influence of treatment methods on the level of exercise capacity in children and sedentary behaviors.

## 2. Materials and Methods

### 2.1. Study Group

A total of 30 children were recruited in the research. Four children refused to take part in the cardiorespiratory fitness test. Two children manifested the symptoms of prolonged malaise. Moreover, on the research day, in three children, the level of platelet count was too low to take physical effort.

The study was comprised of 21 children (12 boys, 9 girls) undergoing treatment for ALL (*n* = 13) and acute myeloid leukemia (AML) (*n* = 8). The subjects were aged 7–13 years (mean age 10.7, SD 2 years; mean height 144, SD 16 cm; mean weight 41.28, SD 13.82 kg) (Table 1).

The subjects were also divided into two separate groups. The criterion for the division was the age of children (7–10 years and 11–13 years). Separate descriptive statistics were used in the groups (Table 2). Children undergoing treatment for cancer disease were examined. The subjects were the patients of the Department of Pediatric Bone Marrow Transplantation, Oncology and Hematology at the University Hospital in Wroclaw, Poland. All children underwent cycles of chemotherapy which were administered in hospital settings. The mean treatment duration was 6.19 (SD, 1.63 months). No comorbidities were reported in patients such as pulmonary disease, musculoskeletal disease, osteoarthritis, or psychiatric disease.

Based on many specific tests, including cytological assessment, immunophenotyping, and genetic and molecular evaluation, children were qualified for treatment with the AIEOP-BFM ALL 2017 protocol or the AML-BFM 2012 protocol. Children treated for ALL (*n* = 13) were included in the AIEOP-BFM ALL 2017 protocol, while those (*n* = 8) treated for AML were included in the AML-BFM 2012 protocol. Depending on the type of ALL, the treatment was different according to the B-ALL (*n* = 7) or the T-ALL regimen (*n* = 6). All children after the first stage of treatment, i.e., after induction, were enrolled in three risk groups based on the following criteria: age, leukocyte count, type of leukemia, treatment response rate, remission, and cytogenetic results. Six children were enrolled in the standard risk (SR) group and 3 children in the intermediate risk (IR) group. The largest group included children who were enrolled in the high risk (HR) group (*n* = 12). In the study group, 13 subjects were given glucocorticoids during treatment, while 8 children were not given such drugs, which was due to the fact that different treatment protocols were used in these groups, depending on the type of the disease (ALL or AML).

### 2.2. Research Methods

#### 2.2.1. Cardiorespiratory Fitness

In the initial stage of the study, anthropometric measurements were performed in each subject (height and weight). Cardiorespiratory fitness was assessed using the ergospirometry test (CPET). Progressive Godfrey protocol was performed [19]. The exercise stress test using the ergospirometer was initiated with a 3 min warm-up at 15 W (height of 120–150 cm) or 20 W (height > 150 cm). Body height >120 cm resulted in proper selection of the cycloergometer for a child.

− After a warm-up period, which prepared the body for greater effort, the test started. During the main part of the test, the load was increased at 1 min intervals by 15 or 20 W (depending on the height of the subject). During the exercise stress test, the pedal frequency was constant (60–80 rotations per minute; RPM). The peak value of exercise was defined as the moment when one of the three criteria was met and the test was then interrupted;
− The decrease in pedal frequency < 60 RPM despite the strong verbal encouragement of the researcher;
− HR_peak_ > 180 beats per minute (bpm);
− Peak respiratory exchange ratio (RER_peak_) > 1.0.

Table 2 shows the mean peak values of HR and RQ. However, these values were not always the main criteria for the termination of the test. The level of RPM below 60 was often the criterion for terminating the test [20].

The VO_2peak_ value was adopted as the mean value from the last 30 s of the exercise stress test [20]. Due to safety reasons, the study did not assess the maximum oxygen uptake in children (VO_2max_), but only the peak value of this parameter (VO_2peak_). The measured VO_2peak_ values were compared with the predicted values for age and sex of the subjects [21].

The measurements of VO_2_ and VCO_2_ allowed the determination of the respiratory quotient (RQ) of the subjects and enabled the calculation of the physiological cost of physical exercise: RQ = VCO_2 exhaled_/VO_2 uptake._

The enrollment was done by the attending physician. Moreover, each parent/legal guardian consented to the participation of the child in the study. The inclusion and exclusion criteria were defined (Table 3). Appropriate selection of the age group (7–13 years) resulted in the elimination of puberty-related factors, which could influence the examined parameters. During puberty, the exercise capacity parameters were significantly different between the groups of boys and girls.

The ergospirometry system (K4b2; COSMED) was used to assess the exercise capacity parameters. This portable system is used for the measurement of pulmonary gas exchange and indirect calorimetry (VO2, VCO2, and RQ). Ergospirometer allows the measurement of O_2_ and CO_2_ concentrations during both inspiratory and expiratory phases.

Cycloergometer ASPEL CRG200 was used during the test. It allowed us to set the appropriate load at the programmed time intervals. The cycloergometer is designed for use with the CardioTEST stress test system and the AsTER cardiac rehabilitation system.

#### 2.2.2. Subjective Assessment of the Physical Activity Level

Anonymous questionnaire surveys were conducted in the traditional paper form. Children who had problems with understanding the text due to age or those who could not assess the time properly were provided with the assistance of their parents or guardians to complete the survey.

The level of physical activity of the subjects was assessed by using the questions from the Health Behavior in School-Aged Children (HBSC 2018) questionnaire from the section connected with health behavior. The questions were related to the last seven days. In the beginning, we assessed what physical activity was and the characteristics of vigorous physical exercise that are the activities and tasks during which the subject experienced an increased heart rate, increased respiratory rate, or temporary breathlessness. Moderate-to-vigorous physical activity (MVPA) was assessed (i.e., the number of days a week during which children exercised for at least 60 min). Additionally, the frequency of undertaking vigorous physical activity was also assessed. The questionnaire included three questions related to sedentary behaviors, i.e., the time in a sitting position in front of a TV or computer screen, time devoted to computer/console stationary games except for movement-related games, time devoted to Internet use to contact the peers and to do homework, etc.

### 2.3. Ethics

The study was approved by the Local Bioethics Committee at the University of Physical Education in Wroclaw, Poland (consent no 22/2018).

### 2.4. Statistical Analysis

Statistical analysis was performed in the GraphPad Prism 7 (Institute of Immunology and Experimental Therapy, Wroclaw, Poland). Normality of the data distribution was assessed by using the Shapiro–Wilk test. The parameters determining the characteristics of the study group were presented by providing the descriptive statistics, including the arithmetic mean and the standard deviation. The Student’s *t*-test for independent groups (unpaired *t*-test) was used to assess the statistical significance of the differences in the results between the study group and the predicted values for age and sex.

The assessment of the relationship between exercise capacity and the applied treatment protocol was checked by using the Student’s *t*-test for independent variable pairs. A one-way ANOVA analysis of variance was used to assess the relationship between the level of physical capacity and the treatment regimen. It was used to compare the three regimens. The Bartlett test was applied to assess the homoscedasticity of the variables.

The assessment of the relationship between the level of exercise capacity and the risk group (to which each child included in a different treatment regimen was enrolled) was conducted. For this purpose, the Spearman’s rank correlation was used. Moreover, the results of the exercise stress test were divided into subgroups according to the risk group and the Student’s *t*-test for independent variable pairs was performed. Moreover, a non-parametric analysis of variance (ANOVA), i.e., the Kruskal–Wallis test for all three subvariables was performed.

The results of the HBSC surveys were presented as percentage data indicating the number of answers to particular questions. The values of the correlation of the survey results that assessed the level of physical activity and sedentary behaviors with the parameters of blood count and the heart rate were analyzed by means of the Spearman’s rank correlation due to the lack of normality of the variable distribution. The significance level at *p* < 0.05 was used.

## 3. Results

### 3.1. Cardiorespiratory Fitness

**Figure 1:**
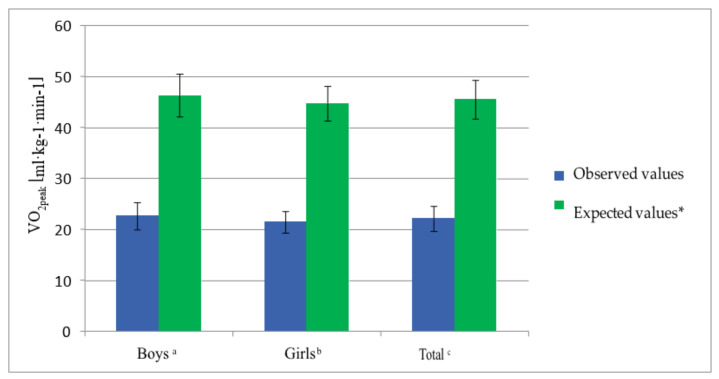
Measured values of VO_2peak_ compared to the predicted values for age and sex in boys, girls and the group in total. Unpaired *t*-test: ^a^ examined boys versus expected values *p* < 0.0001, ^b^ examined girls versus expected values *p* < 0.0001, and ^c^ general study group versus expected values *p* < 0.0001. * Based on age- and sex-predicted values [21].

The measured value of VO_2 peak_ in the group of children was, on average, 22.16 mL·kg^−1^·min^−1^ (SD, 2.5). In the group of boys, this parameter was 22.67 mL·kg^−1^·min^−1^ (SD, 2.7), whereas, in the study group of girls, it was 21.49 mL·kg^−1^·min^−1^ (SD, 2.1) (Table 1). The mean value of the VO_2peak_ predicted for this age group was 45.48 mL·kg^−1^·min^−1^ (SD, 3.8). The predicted value of VO_2peak_ in the group of healthy boys was 46.3 mL·kg^−1^·min^−1^ (SD, 4.2), whereas in the group of healthy girls it was 44.7 mL·kg^−1^·min^−1^ (SD, 3.4) (Figure 1).

The absolute difference between the measured and predicted VO_2peak_ between the groups was 23.32 mL·kg^−1^·min^−1^. In the groups of boys and girls, the difference was 23.63 and 23.21 mL·kg^−1^·min^−1^, respectively (Figure 1).

**Figure 2:**
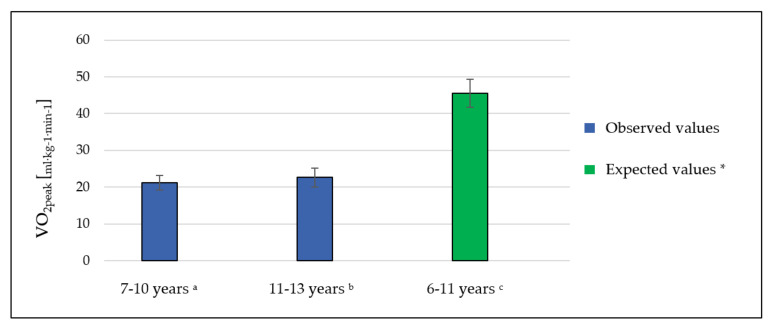
The values of VO_2peak_ in the study group with the division into age groups (children aged 7–10 and 11–13 years) compared to the values predicted in the relevant age group. Unpaired *t*-test: ^a^ examined boys versus expected values *p* < 0.0001, ^b^ examined girls versus expected values *p* < 0.0001, and ^c^ general study group versus expected values *p* < 0.0001. * Based on age- and sex-predicted values [21].

The measured value of VO_2peak_ in the study group with the division into age groups was, on average, 21.21 mL·kg^−1^·min^−1^ (SD, 2.0) in children 7–10 years of age. In the group of children aged 11–13 years, this parameter was 22.64 mL·kg^−1^·min^−1^ (SD, 2.6) (Table 2). The mean values of VO_2peak_ predicted for this age group were 45.48 mL·kg^−1^·min^−1^ (SD, 3.8) (Figure 2).

**Figure 3:**
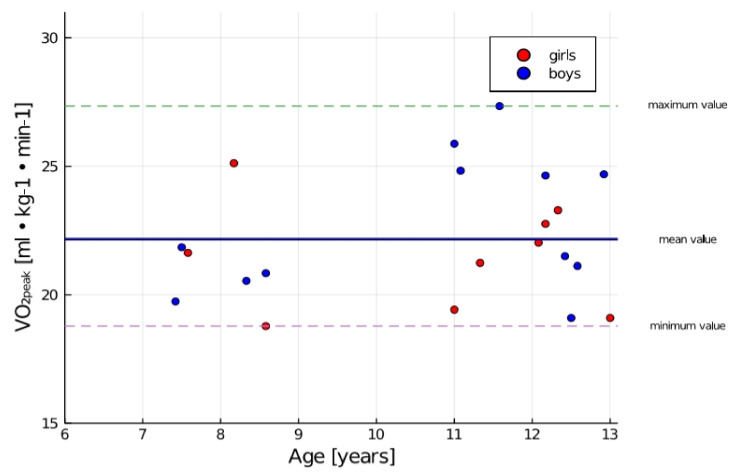
Distribution of measurement results of VO_2peak_ in the group with the division into sex.

In the study group, 38% of children achieved above-average results. The value of VO_2peak_ below 20 mL·kg^−1^·min^−1^ was obtained by 24% of the examined children (Figure 3).

The assessment of the relationships between VO_2peak_ levels of the children and the treatment protocol (ALL-AIEOP-BFM ALL 2017 protocol; AML-AML-BFM-2012 protocol) did not show statistical significance (Table 4).

The assessment of the relationships between the level of exercise capacity (VO_2peak_) and the treatment regimen (B-ALL, T-ALL, and AML-BFM) showed no statistically significant correlations (Table 5).

The assessment of the relationship between the level of exercise capacity (VO_2peak_) and the risk group, including each child (SR, standard risk; IR, intermediate risk; and HR, high risk) who underwent a different treatment regimen, did not show statistically significant values (B-ALL, T-ALL, and AML-BFM) (Table 6).

The study showed that physical exercise at the end of the exercise stress test was vigorous. This is evidenced by the high respiratory quotient (RQ) achieved in the last 30 s of the test. The mean RQ in the study group was 0.93 (SD, 0.24) (Table 1). The study results also showed that children had low blood counts, including hemoglobin (Hb) level (mean 8.34, SD 0.18) (Table 1). High RQ correlated with low hemoglobin levels (Table 7).

The analysis of the relationship of the results of the exercise stress test with the division into age groups showed a positive relationship between the level of the minute ventilation (VE) and the number of WBC in children aged 11–13 years. An inversely proportional relationship was observed in the correlation between high RER values and low RBC in the group of children aged 7–10 years. The same relationship was observed in the correlation between high values of exhaled carbon dioxide (VCO_2_) and VE and low WBC in children aged 11–13 years (Table 8).

### 3.2. Physical Activity Level

None of the children met the standards for the recommended level of weekly physical activity. All subjects declared that during the previous week they did not undertake any form of physical activity longer than 60 min per day (MVPA index). None of the children underwent any moderate or vigorous physical activity in the previous week, i.e., the one that would influence the values of exercise capacity parameters. The percentage of children treated for cancer who declared spending at least 5 h per day in the week in front of a computer or TV screen was 95.24. On days off, all children spent at least 5 h daily in front of the screen. All the children declared playing stationary games daily which did not require physical activity (computer, console, smartphone, or tablet games). These games were only stationary, not active, such as Kinect. On days off, the amount of time spent on games increased. All children declared that they had used the Internet every day and spent at least 4 h daily on it (Table 9).

Table 10 shows the relationship between the level of physical activity (HBSC questionnaire) and morphotic parameters of blood and heart rate. No statistically significant results were observed that could indicate the effect of insufficient level of morphotic parameters of blood on exercise frequency, exercise time, or sedentary behaviors (Table 10).

A positive relationship was found between the amount of time spent using a computer and mobile devices and low WBC in the group of children aged 11–13 years (Table 11).

Table 12 shows the relationship between the level of exercise capacity parameters obtained in the ergospirometry test and the levels of physical activity and sedentary behaviors. There was no effect of reduced physical activity or increased sedentary behaviors on the exercise capacity on children treated for leukemia (Table 12).

The analysis of the relationships between the level of physical activity and the results obtained in the exercise stress test with the division into age groups (7–10 and 11–13 years of age) did not show statistically significant observations (Table 13).

## 4. Discussion

The results of the study showed reduced peak oxygen uptake, which indicates reduced exercise capacity in children during leukemia treatment. The VO_2peak_ parameter was assessed in the study because, in the group of children, it is particularly difficult to examine the effort at the maximum level. Moreover, reaching the plateau is often impossible despite the increase in exercise load. In children, the values of oxygen uptake before reaching the plateau are assumed to be the maximum values. In addition, cancer itself and its sequelae are contraindications for performing maximum tests [23,24]. The evaluated VO_2peak_ values were lower, on average, by 23.32 mL·kg^−1^·min^−1^, compared to healthy children in a similar age group [21].

Children achieved high RQ values during the measurement, which indicates that the level of physical effort was high. Relatively low HR peak values were also observed when the children completed the test (139.5 bpm; SD, 21.32) (Table 1). The duration of the test was relatively short (mean time, 470.67 s; SD, 33.91), and children often interrupted the test prematurely and showed the signs of heavy exercise load. High RQ values correlated with low hemoglobin levels (Table 7), which indicates impaired oxygen transport to muscles. These results suggest that children prematurely felt the symptoms of fatigue, confirming the reduced exercise tolerance of the study subjects.

Unexpectedly, the results of our study also showed that the VO_2peak_ values were significantly lower compared to the results of other researchers who evaluated the level of exercise capacity in children with cancer. The difference was 9.54 mL·kg^−1^·min^−1^ (SD, 9.2). One of the possible reasons for such a difference may be related to a different age group. Braam et al., in their study, assessed the level of exercise capacity in the group of children aged 8–18 years. A large group included children in the puberty period [17]. Intensive changes occur at puberty, and the exercise capacity parameters show significantly different values in boys and girls. Our study results were related to a narrow age group of children who were mostly before puberty (mean age, 10.7; SD, 2.0). This allowed the elimination of the influence of puberty-related factors on the VO_2peak_ parameter [25].

Another reason for such a large discrepancy in the results obtained may be related to the different stage of cancer treatment. The study subjects were at the initial stage of cancer treatment. The mean treatment time was 6.0 months (SD, 2.0) in the study group. In the early stage of cancer disease, the therapy is very aggressive and is aimed at the fastest destruction of a large number of cancer cells. One of the adverse effects of the first stage of cancer treatment is related to the adverse effect on morphotic parameters of blood. Anemia is a particularly common symptom. A decreased hemoglobin level is the cause of insufficient oxygen transport to muscles. As a result, it is manifested by a decrease in exercise capacity in children [26]. Our study included only children treated for leukemia (ALL *n* = 13; AML *n* = 8). This disease of the hematopoietic system also directly adversely affects the morphotic parameters of blood and determines the exercise capacity [27].

Cancer treatment is also the cause of adverse changes that affect the circulatory system. Chemotherapy often has a cardiotoxic effect and can cause heart failure. It was also a limiting factor for children to undertake full-time physical activity. Our exercise stress tests were conducted in a group of children without circulatory disorders. Symptoms such as dyspnea, coughing, and fainting may indicate cardiorespiratory abnormalities resulting from the adverse effects of chemotherapy [28].

Cachexia is a common sequela of cancer, which is manifested by weight loss and muscle atrophy. It has a negative effect on the exercise capacity in children undergoing treatment for cancer [29,30]. This reduces the exercise capacity level, which is still insufficient in the group of childhood cancer survivors. Low muscle mass and the deficiency of energy substrates are also important factors that have a direct impact on the reduction in exercise capacity [31].

Low blood counts and insufficient oxygen uptake are also manifested by general malaise and excessive fatigue in children. This increases the reluctance to undertake physical exercise and sometimes even the fear of the occurrence of fatigue symptoms, such as fainting, increased sweating, and headaches. Lack of contact with peers and being surrounded only by adults affect children’s motivation to be active and their bad mental state. Most children declared that they had not undertaken any form of physical activity in the previous week (Table 9).

One of the important factors influencing the final result of the cardiopulmonary exercise test is the level of motivation of the examined subjects. Children with cancer disease have a low level of motivation. It is not only due to the disease and the related procedures, which often cause fear and anxiety. Additionally, psychosocial problems resulting from cancer treatment adversely influence motivation in children. Parental attitude and excessive care are the reasons for low motivation in children to undertake physical activity out of their own will. This may result in a worse perception of the child’s ability to undertake physical activity. As a result, it causes a premature termination of the stress test despite the absence of fatigue symptoms [32].

None of the children reported any form of physical activity in the previous week; nor did they meet the MVPA recommendations (Table 9). It is also a possible cause of achieving low VO_2peak_ values.

It can be assumed that one of the reasons for the lack of interest in sports and physical activity is the lack of attractive forms of physical activity dedicated to children during hospitalization.

Children with no functional deficits who do not require special rehabilitation are not offered any form of physical activity. Promoting attractive forms of physical effort (e.g., exercise using virtual reality technology or interactive games) as part of cancer therapy may prevent a significant decrease in exercise capacity. Such forms of exercise would positively influence improving and maintaining exercise capacity parameters [33].

Habits acquired during treatment often become permanent and remain even after treatment. Reduced VO_2peak_ is an important predictor of premature mortality. Therefore, it is important that children treated for cancer should undertake physical activity and maintain high values of VO_2peak_ [10,34,35].

In conclusion, it is important to prevent excessive exercise capacity deficits and to maintain physical activity at a level adapted to the current abilities of the treated children from the onset of cancer treatment. Lack of physical activity during cancer treatment remains a habit and a factor predisposing this group of patients to the occurrence of diseases of affluence in adult life. Rehabilitation of children with cancer is particularly significant at the hospital stage. However, a frequent problem is related to the selection of appropriate forms of exercise for these children and the selection of appropriate exercise intensity in particular. The results of the exercise stress test are a very useful tool to help plan an individual rehabilitation program for a child with cancer. The comparison of the results of the exercise stress test in the group of affected children with the results of healthy children is a strong motivator to undertake action to increase the level of physical activity of these children, which in turn will improve their level of exercise capacity.

## 5. Strengths and Limitations of This Study

The examined group of children was very homogeneous in terms of age, treatment time, the type of disease, and treatment procedures. The methods used to measure respiratory parameters, including the parameter assessing exercise capacity in children (VO_2peak_), were objective. However, these methods are rarely used in the group of children during cancer therapy. This is probably due to the difficult access to a homogeneous study group and frequently aggressive medical procedures applied to children. Consequently, the duration of the entire research period and the time of data collection are often very long. A large number of children who could be included in the study group did not agree to participate in the study, due to the high intensity of chemotherapy and fatigue or malaise symptoms. Sometimes low PLT is a contraindication for cardiopulmonary stress testing. Taking these factors into account, we assert that the study group is relatively large. In terms of statistical analysis, the study group was small (*n* = 21), which could increase the risk of statistical error and undermine the inference.

In some cases, CPET could have lasted longer and the achieved VO_2peak_ values could have been higher. A frequent reason for interrupting the cardiopulmonary exercise test was the lack of motivation among children. The subjects undergoing cancer treatment showed a premature desire to terminate the test despite low heart rate and RQ values. Despite the researcher’s encouragement, the subjects significantly reduced the pedaling frequency. Moreover, the study coincided with the COVID-19 pandemic, which resulted in the suspension of the study. The study group included the patients from the high-risk group of infection, which made it impossible to conduct the study in its complete scope. In the future, the study period should be prolonged, and the study should be conducted on a larger group of children with cancer. Moreover, the methods used for the assessment of the level of physical activity were not objective. These methods only estimated the weekly level of physical activity of children. Therefore, the use of devices such as Sport Tester or ActiGraph would certainly provide objective data.

## 6. Future Research Directions

The assessment of the baseline level of the exercise capacity should be the starting point for the whole process of rehabilitation of a child with cancer. In the future, the test results will serve as a prognostic element enabling individual selection of the intensity of the exercise/rehabilitation program. This will enable the maintenance of exercise-capacity parameters and prevent an excessive decrease in the child’s exercise capacity during the long-term process of cancer treatment. Rehabilitation focused on increasing the level of daily physical activity and preventing sedentary behaviors is of crucial importance. However, it is not always properly selected.

## 7. Conclusions

The study results showed that children undergoing treatment for leukemia were characterized by a reduced level of exercise capacity. Their level was much lower compared to healthy children of the same age. The low levels of exercise capacity parameters were probably caused by the medical procedures and many circles of chemotherapy that children underwent. Cancer and its aggressive treatment result in a decrease in the level of morphotic parameters of blood, which results in tissue hypoxia and a significant decrease in exercise capacity parameters. Lack of physical activity and failure to meet the standards for the minimum level of weekly physical activity (MVPA) probably contributed to the deterioration in exercise capacity in children treated for cancer.

Psychosocial factors, such as lack of contact with peers, feeling of isolation, and awareness of a life-threatening disease, contributed to physical inactivity.

## Figures and Tables

**Table 1:** Patient characteristics by sex.

Variables	All Participants *n* = 21		Boys *n* = 12		Girls *n* = 9	
	Mean	SD	Mean	SD	Mean	SD
Determinant variables						
Age (years)	10.7	2.0	10.7	2.1	10.7	2.0
Height (cm)	144.0	16.0	147.0	18.0	141.0	13.0
Weight (kg)	41.28	13.82	42	14.99	40.32	12.91
Treatment duration (months)	6.0	2.0	6.0	2.0	6.0	1.0
HGB (g/dL) *	8.34	0.18	8.32	0.18	8.36	0.19
PLT (G/L) *	157.3	66.15	135.8	71.59	186	47.69
RBC (T/L) *	3.49	0.3	3.46	0.32	3.55	0.29
WBC (G/L) *	2.1	0.96	2.29	1.19	1.84	0.49
HR (at rest) *	85.67	3.812	85.58	4.48	85.78	2.95
Outcome variables						
VO_2peak_ (mL·kg^−1^·min^−1^) *	22.16	2.46	22.67	2.67	21.49	2.11
HR_peak_ *	139.5	21.32	142	24.15	136.1	17.65
VO_2_ (mL·min^−1^) *	1628	2277	1244	293.2	2140	3511
VCO_2_ (mL·min^−1^) *	1130	453.4	1284	461.2	924.9	372.3
VE (L·min^−1^) *	28.94	7.65	30.6	6.43	26.72	8.92
VE/VCO_2_*	27.73	4.99	26.02	4.51	30	4.91
RQ = VCO_2 exhaled_/ VO_2 uptake_ *	0.93	0.24	1.0	0.17	0.82	0.29
MET *	6.28	0.7	6.4	0.76	6.1	0.6
Test duration (s)	470.67	33.91	471.5	35.75	469.55	31.26

* HGB, hemoglobin level; PLT, blood platelet count; RBC, red blood cell count; WBC, white blood cell count; HR (at rest), resting heart rate; VO_2peak_,peak oxygen uptake; HR_peak_, peak heart rate; VO_2_, volume of O_2_ uptake; VCO_2_, volume of exhaled CO_2_; VE, minute ventilation; VE/VCO_2_,ventilatory equivalent of carbon dioxide; RQ, respiratory quotient; MET, metabolic equivalent of task.

**Table 2:** Patient characteristics by age group (7–10 and 11–13 years of age).

Variables	7–10 Years *n* = 7		11–13 Years *n* = 14	
	Mean	SD	Mean	SD
Determinant variables				
Age (years)	8.0	0.5	12.0	0.7
Height (cm)	128.0	6.0	152.0	12.0
Weight (kg)	30.13	5.156	46.86	13.46
Treatment duration (months)	6.429	1.512	6.071	1.73
HGB (g/dL) *	8.357	0.1902	8.329	0.1773
PLT (G/L) *	166	52.05	153	73.63
RBC (T/L) *	3.68	0.2312	3.409	0.2999
WBC (G/L) *	1.811	0.4956	2.245	1.118
HR at rest *	87.71	2.215	84.64	4.088
Outcome variables				
VO_2peak_ (mL·kg^−1^·min^−1^) *	21.21	2.022	22.64	2.593
HR_peak_ *	151	6.325	152.5	12.97
VO_2_ (mL·min^−1^) *	2667	3893	1108	331.6
VCO_2_ (mL·min^−1^) *	1255	538.2	1068	412.3
VE (L·min^−1^) *	29.81	7.71	28.5	7.867
VE/VCO_2_ *	25.63	5.56	28.77	4.525
RQ = VCO_2 exhaled_/ VO_2 uptake_ *	0.891	0.3968	0.9465	0.1341
MET *	6.0	0.5715	6.414	0.742
Test duration (s)	458.4	35.59	476.8	33.95

* HGB, hemoglobin level; PLT, blood platelet count; RBC, red blood cell count; WBC, white blood cell count; HR (at rest), resting heart rate; VO_2peak_, peak oxygen uptake; HR_peak_, peak heart rate; VO_2_-volume of O_2_ uptake; VCO_2_, volume of exhaled CO_2_; VE, minute ventilation; VE/VCO_2_, ventilatory equivalent of carbon dioxide; RQ, respiratory quotient; MET, metabolic equivalent of task.

**Table 3:** Inclusion and exclusion criteria in the study group.

Inclusion Criteria	Exclusion Criteria
- Diagnosed cancer disease: ALL or AML; - Hospital treatment; - Duration of hospitalization > 7 days; - Chemotherapy; - No physical disability, unassisted arrival at the examination; - Written informed consent of the parent/legal guardian for the participation in the study; - Age: 7–13 years, height > 120 cm.	- Platelet count < 20 G/L; - Hemoglobin concentration < 8 g /dL; - Infectious disease with fever > 38 °C; - Intellectual disability.

**Table 4:** The relationship between the level of exercise capacity (VO_2peak_) and the treatment protocol (AIEOP-BFM ALL 2017; 1-AML-BFM-2012).

Variables	VO_2peak_ Treatment Protocol (AIEOP-BFM ALL 2017), *n* = 13	VO_2peak_ Treatment Protocol (AML-BFM-2012), *n* = 8
VO_2peak_ treatment protocol (AIEOP-BFM ALL 2017)	-	-
vs.	-	-
VO_2peak_ treatment protocol (AML-BFM-2012)	-	-
Unpaired *t*-test		
*p*-value		0.5149
*p*-value summary	Ns	
Significantly different (*p* < 0.05)?		No
One-or two-tailed *p*-value?	Two-tailed	

**Table 5:** Relationship between the level of exercise capacity (VO_2peak_) and the treatment regimen (B-ALL, T-ALL, and AML-BFM).

Variables	Statistical Values
VO_2peak_ treatment regimen (B-ALL), *n* = 7	
vs.	
VO_2peak_ treatment regimen (T-ALL), *n* = 6	
vs.	
VO_2peak_ treatment regimen (AML-BFM), *n* = 8	
One-way ANOVA	
F	0.1775
*p*-value	0.8388
*p*-value summary	Ns
Significant difference among means (*p* < 0.05)?	No
R square	0.01934
Bartlett’s test for homoscedasticity	
Bartlett’s statistic (corrected)	0.1198
*p*-value	0.9419
*p*-value summary	Ns
Are SDs significantly different (*p* < 0.05)?	No

**Table 6:** The relationship between the level of exercise capacity (VO_2peak_) and the risk group (SR, standard risk; IR, intermediate risk; and HR, high risk).

Variables	Treatment Regimen	Risk Group SR: *n* = 6, HR: *n* = 3, IR: *n* = 12	VO_2peak_
Spearman Rho	Treatment regimen	Risk group	VO_2peak_
Treatment regimen		−0.388	−0.268
Risk group	−0.388		−0.011
VO_2peak_	−0.268	−0.011	
*p*-values	Treatment regimen	Risk group	VO_2peak_
Treatment regimen		0.082	0.241
Risk group	0.082		0.962
VO_2peak_	0.241	0.962	
VO_2peak_ _risk group–high risk			
vs.			
VO_2peak__risk group-standard risk			
Unpaired *t*-test			
*p*-value	0.9881		
*p*-value summary	Ns		
Significantly different (*p* < 0.05)?	No		
One-or two-tailed *p*-value?	Two-tailed		
t, DF	t = 0.01521 DF = 16		
Kruskal–Wallis test of VO_2peak__risk group (SR, IR, HR)			
*p*-value	0.6766		
Exact or approximate *p*-value?	Exact		
*p*-value summary	Ns		
Do the medians vary significantly (*p* < 0.05)?	No		
Number of groups	3		
Kruskal–Wallis statistic	0.8447		

**Table 7:** Correlation of the results of the exercise stress test with the parameters of blood count and heart rate in the study group of children in total. Values of *p* < 0.05 were considered statistically significant.

Spearman Rho	Hb (mg/dL)	PLT (G/L)	RBC (T/L)	WBC (G/L)	HR at Rest	Exercise HR
VO_2peak_ (mL·kg^−1^·min^−1^)	−0.22	−0.15	−0.09	−0.02	0.22	0.25
VO_2_ (mL·min^−1^)	0.11	−0.12	−0.09	0.01	0.16	−0.02
VCO_2_ (mL·min^−1^)	−0.01	−0.18	−0.10	−0.03	0.09	−0.09
RER	−0.28	−0.01	−0.09	−0.20	0.23	−0.27
VE (L·min^−1^)	0.12	−0.03	−0.22	0.14	0.11	0.08
VE/VCO_2_	−0.06	0.25	−0.15	0.24	0.09	0.11
RQ = VCO_2 exhaled/_VO_2 uptake_	−0.45 *	−0.12	−0.14	−0.24	0.13	−0.30
MET	−0.21	−0.16	−0.11	−0.03	0.21	0.23

* Results showing the correlation.

**Table 8:** Correlation between the exercise stress test results and blood count parameters and heart rate with the division into age groups (7–10 and 11–13 years). Values of *p* < 0.05 were considered statistically significant.

Spearman Rho	Hb (mg/dL)		PLT (G/L)		RBC (T/L)		WBC (G/L)		HR at Rest		Exercise HR	
	I	II	I	II	I	II	I	II	I	II	I	II
VO_2peak_ (mL·kg^−1^·min^−1^)	−0.44	−0.13	−0.32	−0.15	−0.20	0.01	−0.25	−0.05	0.64	0.20	−0.36	0.39
VO_2_ (mL·min^−1^)	0.44	−0.03	0.36	−0.45	−0.61	−0.15	−0.61	0.41	0.15	0.14	0.34	−0.11
VCO_2_ (mL·min^−1^)	0.00	0.01	0.14	−0.40	−0.68	−0.06	−0.93 *	0.46	0.13	0.10	0.07	−0.12
RER	−0.44	−0.36	0.24	−0.25	−0.93 *	0.17	−0.73	0.18	−0.18	0.37	−0.23	−0.36
VE (L·min^−1^)	0.16	0.08	0.00	−0.28	−0.68	−0.19	−0.79 *	0.56 *	0.11	0.11	−0.02	0.04
VE/VCO_2_	0.16	−0.14	−0.14	0.39	0.47	−0.21	0.75	−0.03	0.20	0.19	−0.18	0.09
RQ = VCO_2 exhaled/_VO_2 uptake_	−0.76	−0.35	−0.14	−0.26	−0.58	0.19	−0.57	0.17	−0.25	0.36	−0.45	−0.36
MET	−0.44	−0.11	−0.32	−0.15	−0.20	−0.02	−0.25	−0.06	0.64	0.19	−0.36	0.37

* Results showing the correlation. Group I = children aged 7–10; group II = children aged 11–13.

**Table 9:** Level of physical activity and sedentary behaviors. Percentage data on the number of people answering questions from the HBSC survey (%).

Response *	The Number of Days Per Week in Which the Child Performed the Physical Activity of at Least 60 min (MVPA)—HBSC 1	Frequency of Undertaking Vigorous Physical Activity—HBSC 2	The Number of Hours in Front of a Screen Per Week—HBSC 4.1	The Number of Hours in Front of a Screen at the Weekend—HBSC 4.2	The Number of Hours Spent Playing Games Per Week—HBSC 5.1	The Number of Hours Spent Playing Games at the Weekend—HBSC 5.2	The Number of hours Spent Using a Computer, Tablet or Smartphone Per Week—HBSC 6.1	The Number of Hours Spent Using a Computer, Tablet or Smartphone at the Weekend—HBSC 6.2
0	100	-	-	-	-	-	-	-
1	-	-	-	-	-	-	-	-
2	-	-	-	-	-	-	-	-
3	-	-	-	-	-	-	-	-
4	-	-	4.76	-	9.52	9.52	-	-
5	-	-	38.10	23.81	38.10	14.29	38.10	14.29
6	-	100	33.33	42.86	52.38	52.38	61.90	38.10
7	-	-	23.81	33.33	-	23.81	-	47.62

* Content of the response included in the previous paper [22]. The third question was not considered in the current study since the new HBSC 2018 questionnaire was formed with the exclusion of question 3.

**Table 10:** Correlation between the survey results that assessed the level of physical activity and sedentary behaviors and blood count parameters and heart rate in the study group of children in total. Values of *p* < 0.05 were considered statistically significant. ^a^ The question content of the HBSC questionnaire is given in Table 7.

Spearman Rho	Hb (mg/dL)	PLT (G/L)	RBC (T/L)	WBC (G/L)	HR at Rest	Exercise HR
**HBSC 1 ^a^**	-	-	-	-	-	-
**HBSC 2 ^a^**	-	-	-	-	-	-
**HBSC 4.1 ^a^**	0.01	−0.25	0.31	0.15	0.20	−0.01
**HBSC 4.2 ^a^**	0.11	0.07	−0.04	0.32	0.27	0.25
**HBSC 5.1 ^a^**	−0.08	−0.08	0.10	−0.30	0.21	0.04
**HBSC 5.2 ^a^**	−0.06	−0.29	0.13	−0.10	0.01	−0.04
**HBSC 6.1 ^a^**	−0.18	−0.06	0.09	−0.25	-0.15	−0.03
**HBSC 6.2 ^a^**	−0.02	0.28	0.12	−0.46 *	0.31	−0.08

* Results showing the correlation.

**Table 11:** Correlation between the survey results that assessed the level of physical activity and sedentary behaviors and blood count parameters and heart rate with the division into age groups (7–10 and 11–13 years of age). Values of *p* < 0.05 were considered statistically significant. ^a^ The question content of the HBSC questionnaire is presented in Table 7.

Spearman Rho	Hb (mg/dL)		PLT (G/L)		RBC (T/L)		WBC (G/L)		HR at Rest		Exercise HR	
	I	II	I	II	I	II	I	II	I	II	I	II
HBSC 1 ^a^	-	-	-	-	-	-	-	-	-	-	-	-
HBSC 2 ^a^	-	-	-	-	-	-	-	-	-	-	-	-
HBSC 4.1 ^a^	−0.27	−0.42	0.30	0.02	0.21	0.02	0.00	−0.27	0.33	0.10	−0.36	−0.42
HBSC 4.2 ^a^	−0.58	−0.69 *	−0.19	−0.10	0.10	0.00	−0.19	−0.42	0.53	0.24	−0.81 *	−0.25
HBSC 5.1 ^a^	0.24	−0.15	0.39	0.02	−0.12	0.14	0.23	0.33	−0.53	−0.07	0.78	−0.21
HBSC 5.2 ^a^	0.48	−0.27	0.61	−0.03	0.00	−0.16	−0.04	0.55 *	−0.24	0.27	0.87 *	−0.13
HBSC 6.1 ^a^	0.31	−0.45	−0.61	−0.16	0.41	−0.18	0.41	−0.18	0.21	0.16	−0.21	−0.37
HBSC 6.2 ^a^	0.00	−0.45	−0.29	−0.21	−0.22	0.13	−0.58	−0.21	0.29	0.15	−0.51	−0.53

* Results showing correlation. Group I = children aged 7–10; group II = children aged 11–13.

**Table 12:** Correlation between the survey results that assessed the level of physical activity and sedentary behaviors and the results of the exercise stress test in the study group of children in total. Values of *p* < 0.05 were considered statistically significant. ^a^ The question content of the HBSC questionnaire is presented in Table 7.

Spearman Rho	VO_2peak_ (mL·kg^−1^·min^−1^)	VO_2_ (mL·min^−1^)	VCO_2_ (mL·min^−1^)	RER	VE (L·min^−1^)	VE/VCO_2_	RQ = VCO_2 exhaled/_VO_2 uptake_	HR_peak_	MET
HBSC 1 ^a^	-	-	-	-	-	-	-		-
HBSC 2 ^a^	-	-	-	-	-	-	-	-	-
HBSC 4.1 ^a^	0.21	−0.04	−0.05	−0.24	−0.09	0.03	−0.26	−0.26	0.19
HBSC 4.2 ^a^	0.31	0.14	0.02	−0.08	0.15	0.13	−0.21	−0.11	0.30
HBSC 5.1 ^a^	−0.11	0.21	0.26	0.30	0.15	−0.44 *	0.39	0.00	−0.10
HBSC 5.2 ^a^	−0.05	0.30	0.30	0.19	0.11	−0.48 *	0.20	−0.08	−0.04
HBSC 6.1 ^a^	0.36	−0.15	−0.20	−0.10	−0.17	0.18	−0.17	−0.01	0.34
HBSC 6.2 ^a^	0.27	−0.22	−0.30	−0.10	−0.36	0.11	−0.21	−0.01	0.26

* Results showing correlation.

**Table 13:** Correlation between the results of the questionnaire that assessed the level of physical activity and sedentary behaviors and the results of the exercise stress test with the division into age groups (7–10 and 11–13 years of age). Values of *p* < 0.05 were considered statistically significant. ^a^ The question content of the HBSC questionnaire is presented in Table 7.

Spearman Rho	VO_2peak_ (mL·kg^−1^·min^−1^)		VO_2_ (mL·min^−1^)		VCO_2_ (mL·min^−1^)		RER		VE (L·min^−1^)		VE/VCO_2_		RQ = VCO_2 exhaled/_VO_2 uptake_		HR_peak_		MET	
	I	II	I	II	I	II	I	II	I	II	I	II	I	II	I	II	I	II
HBSC 1 ^a^	-	-	-	-	-	-	-	-	-	-	-	-	-	-	-	-	-	-
HBSC 2 ^a^	-	-	-	-	-	-	-	-	-	-	-	-	-	-	-	-	-	-
HBSC 4.1 ^a^	0.00	−0.39	−0.42	−0.12	−0.30	−0.10	−0.27	0.13	−0.54	−0.08	0.18	0.20	−0.12	0.11	−0.24	0.28	0.00	−0.45
HBSC 4.2 ^a^	0.28	0.12	−0.47	−0.19	−0.19	−0.24	−0.10	−0.02	−0.28	−0.25	0.19	0.18	0.19	−0.03	0.00	−0.23	0.28	0.06
HBSC 5.1 ^a^	−0.08	0.04	0.31	0.20	0.08	0.26	0.20	0.22	−0.04	0.10	−0.19	−0.25	−0.08	0.22	0.18	0.37	−0.08	0.05
HBSC 5.2 ^a^	−0.29	−0.07	0.42	0.42	0.26	0.46	0.03	0.16	0.00	0.31	−0.39	−0.21	−0.26	0.15	−0.08	0.28	−0.29	−0.09
HBSC 6.1 ^a^	0.00	−0.18	−0.20	−0.14	−0.41	−0.18	−0.52	−0.14	0.00	−0.28	0.61	0.19	−0.41	−0.14	−0.51	−0.16	0.00	−0.23
HBSC 6.2 ^a^	−0.14	−0.16	0.14	0.07	0.43	0.09	0.15	0.51	0.58	−0.10	−0.29	−0.10	0.29	0.51	0.07	0.27	−0.14	−0.20

* Results showing the correlation. Group I = children aged 7–10; group II = children aged 11–13.
